# c-Myc is regulated by HIF-2α in chronic hypoxia and influences sensitivity to 5-FU in colon cancer

**DOI:** 10.18632/oncotarget.12911

**Published:** 2016-10-26

**Authors:** Liangjing Wang, Meng Xue, Daniel C. Chung

**Affiliations:** ^1^ Department of Gastroenterology, The Second Affiliated Hospital, School of Medicine, and Institute of Gastroenterology, Zhejiang University, Hangzhou, China; ^2^ Gastrointestinal Unit, Massachusetts General Hospital, Harvard Medical School, Boston, Massachusetts, USA; ^3^ Cancer Center, Massachusetts General Hospital, Harvard Medical School, Boston, Massachusetts, USA

**Keywords:** c-Myc, HIF-2α, 5-FU, colon cancer

## Abstract

Colorectal cancers (CRCs) invariably become hypoxic as they enlarge, and this places unique metabolic demands upon the tumor cells. Hypoxic stress can enhance the invasiveness of cancer cells and induce chemoresistance. c-Myc, an oncogene regulated by hypoxia inducible factors (HIFs), plays a critical role in cell proliferation and metabolism. However, the interplay between c-Myc and HIFs and its clinical significance in hypoxic adaptation in CRCs are unknown. We demonstrate that c-Myc mRNA and protein levels in colon cancer cells are induced within 2 h of hypoxic stress (1% O_2_) but are then significantly downregulated when exposed to prolonged hypoxia. In chronic hypoxia (over 8 h at 1% O_2_), HIF-2α but not HIF-1α gradually accumulated in colon cancer cells. Knockdown of HIF-2α increased levels of c-Myc and its downstream target cyclinD1 in chronic hypoxia, indicating that HIF-2α may function to downregulate c-Myc. Chronic hypoxia suppressed the expression of cyclinD1, CDK4, and CDK6, inducing G1 phase block and 5-flurouracil (5-FU) chemoresistance. Overexpression of c-Myc reversed the inhibition of cyclinD1, CDK4, and CDK6, which accelerated the G1/S phase transition under hypoxia and enhanced sensitivity to 5-FU. In contrast, knockdown of c-Myc impaired 5-FU chemosensitivity in colon cancer cells. In summary, HIF-2α plays an important role in regulating the expression of c-Myc in chronic hypoxia, and consequently controls the sensitivity of colon cancer cells to 5-FU treatment in this environment.

## INTRODUCTION

Colorectal cancers (CRCs) are one of the most common cancers worldwide [1]. Chemotherapy is an integral component of the management of advanced disease, but there can be treatment failures due to drug resistance [2]. When solid tumors grow at a faster rate than neovascularization can sustain, regions of low oxygenation develop [2]. Molecular adaptation to this hypoxic environment results in metastasis, cell cycle retardation, resistance to chemotherapy, and a poor prognosis [3].

Hypoxia inducible factors (HIFs) are key elements mediating cellular responses to hypoxia [4]. c-Myc is a critical target gene of HIF [5] and this regulation is isoform specific [6]. HIF-1α disrupts the interaction between c-Myc and its binding partners including Max and Sp1, whereas HIF-2α stabilizes those complexes, in turn promoting the DNA binding of c-Myc [7]. It is believed that HIF-1α can be expressed ubiquitously whereas HIF-2α expression is more limited [8, 9].

The c-Myc oncogene can regulate cellular proliferation, migration and angiogenesis [10]. However, c-Myc expression is not necessarily a negative prognostic factor in cancers. Two clinical studies demonstrated that preoperative neoadjuvant chemotherapy with 5-flurouracil (5-FU) in breast cancer patients or postoperative adjuvant chemotherapy in CRC patients with c-Myc amplification were associated with better therapeutic responses than in those with no amplification. This was accompanied by longer postoperative relapse-free survival [11, 12], suggesting a correlation between c-Myc expression and chemosensitivity. In this study, we identify the regulation of c-Myc by HIF-2α under chronic hypoxic stress and demonstrate the impact on 5-FU chemosensitivity in colon cancer cells.

## RESULTS

### c-Myc is downregulated under chronic hypoxic stress in colon cancer cells

**Figure 1:**
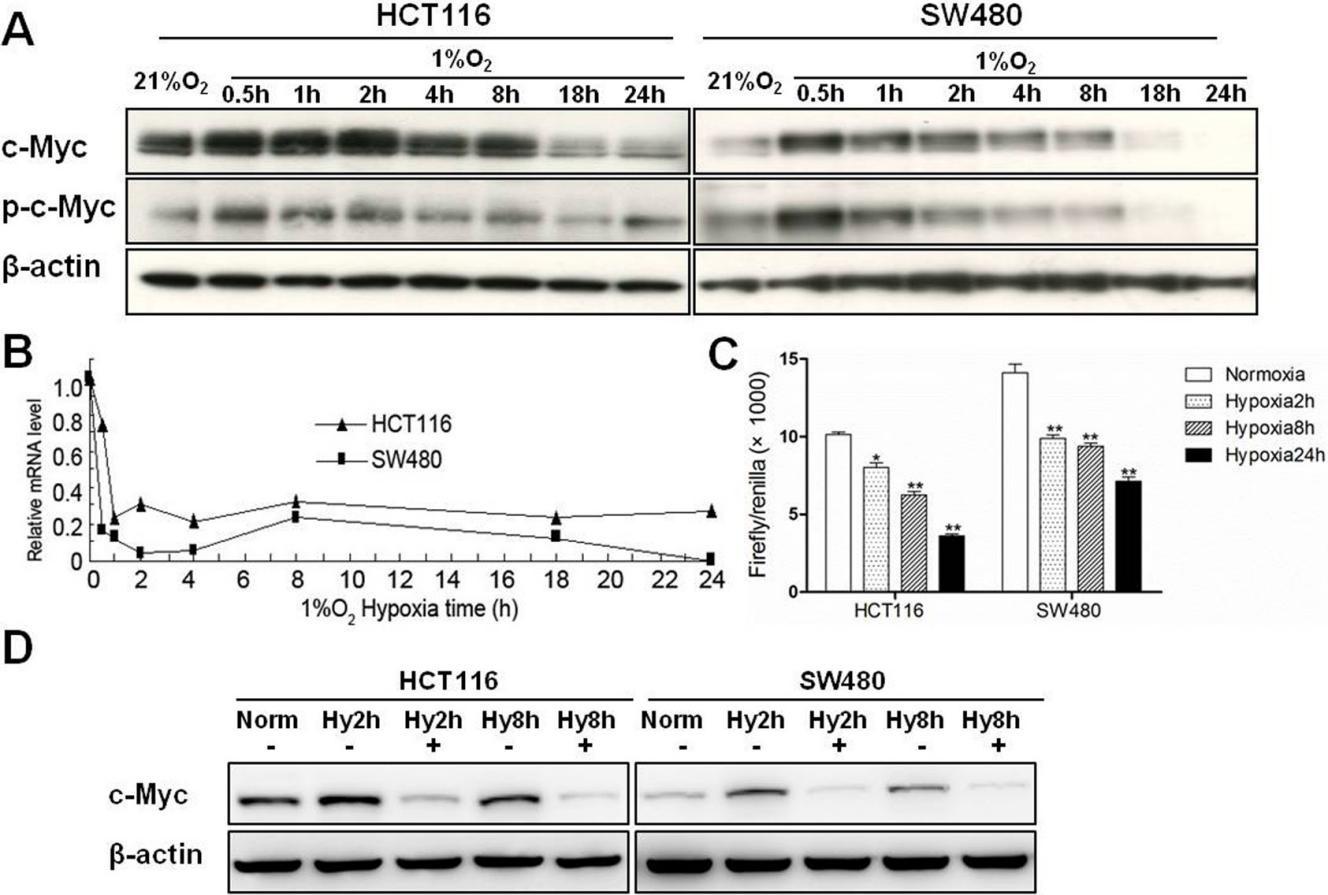
The expression of c-Myc under hypoxic conditions in colon cancer cells (**A**) Total protein and RNA were extracted after HCT116 and SW480 cells were incubated under hypoxic conditions for 0.5 h, 1 h, 2 h, 4 h, 8 h, 18 h and 24 h. Protein levels of c-Myc and phospho-c-Myc in HCT116 and SW480 cells were assessed by western blotting. β-actin was used as an internal control. (**B**) mRNA levels of c-Myc were measured by quantitative real-time PCR. β-actin was used as internal control. (**C**) HCT116 and SW480 cells were co-transfected with pGL3-c-Myc promoter and pRL-TK vector, and cultured in normoxia or hypoxia for 2, 8 or 24 h. Relative firefly luciferase activity (× 1000) was expressed normalized to renilla luciferase activity in pRL-TK vector. Asterisks indicate statistical significance (*P < 0.05, **P < 0.01, vs. Normoxia). (**D**) HCT116 and SW480 cells were treated with 10 μg/ml cycloheximide and cultured under hypoxia for 2 or 8 h. c-Myc protein was measured by Western blotting. All experiments were performed in triplicate.

In order to determine the dynamic expression changes of c-Myc as a function of time in hypoxia, colon cancer cells (HCT116 and SW480) were cultured in normoxic (21% O_2_) or hypoxic (1% O_2_) conditions for 0.5, 1, 2, 4, 8, 18 and 24 hours. Both c-Myc and phosphorylated c-Myc proteins were induced within 2 h of hypoxia but were significantly downregulated when the hypoxic stress exceeded 8 h (Figure 1A). Over the entire time period, there was also a decrease in c-Myc mRNA, and the downregulation was most significant in the first 2 h (Figure 1B). To better understand the transcriptional mechanisms regulating c-Myc by hypoxia, we transfected SW480 and HCT116 cells with a c-Myc promoter luciferase reporter plasmid and then examined luciferase activity at 2, 8 and 24 hours. c-Myc promoter activity in tumor cells was gradually downregulated with the prolongation of hypoxic stress when compared to normoxic conditions (Figure 1C). In addition, the protein levels of c-Myc were remarkably decreased when colon cancer cells were treated with 10 μg/ml cycloheximide, a general inhibitor of protein biosynthesis, and incubated under acute hypoxic conditions (2 and 8 hours) (Figure 1D). Collectively, these results suggest that the initial upregulation of c-Myc protein in hypoxia is primarily post-transcriptional but that chronic hypoxia results in downregulation of c-Myc through reduced transcription.

### c-Myc is predominantly regulated by HIF-2α in hypoxic conditions

**Figure 2:**
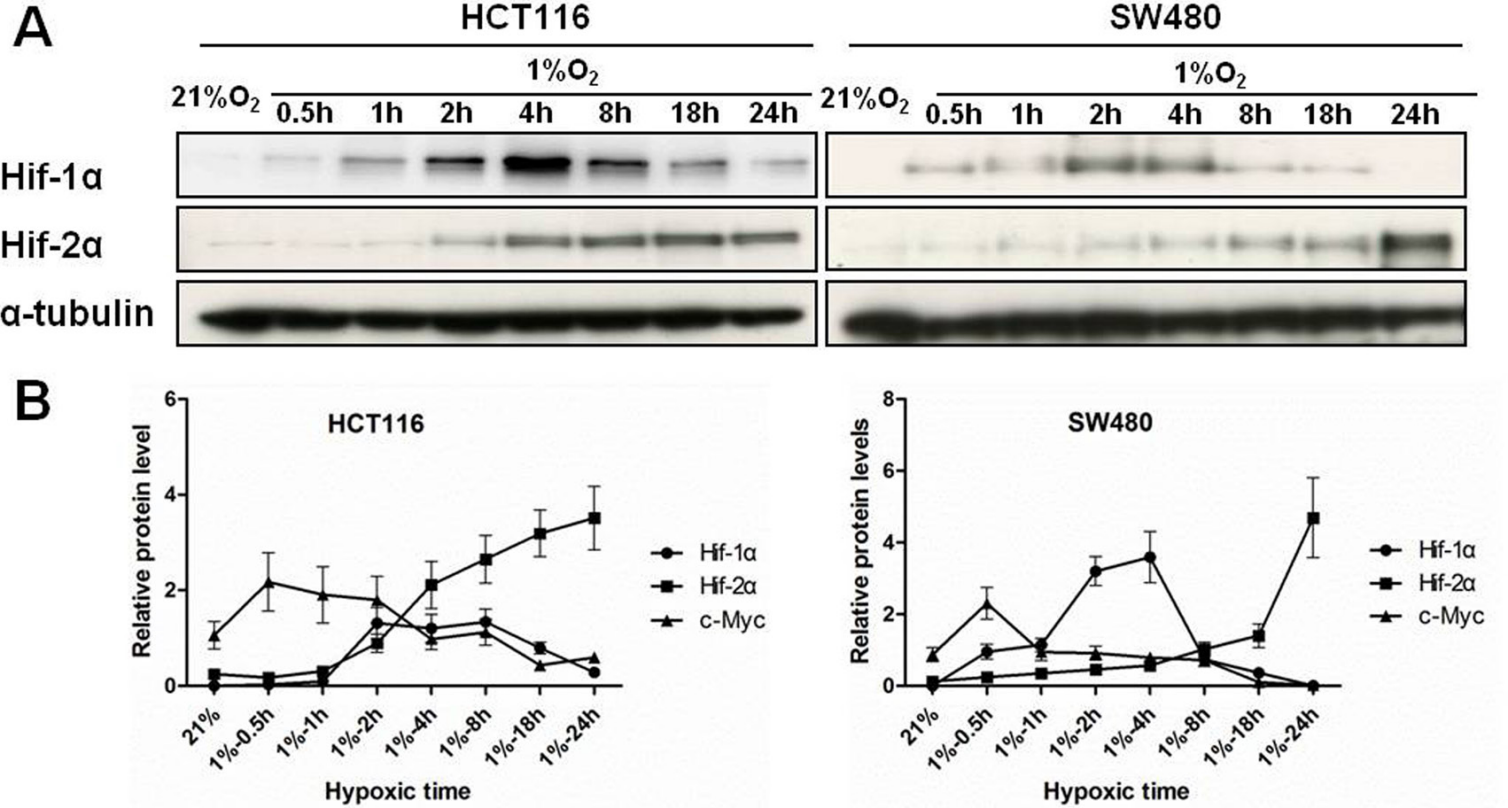
The expression of HIFs and temporal relationship with c-Myc in colon cancer cells (**A**) Total protein was extracted after HCT116 and SW480 cells were incubated under hypoxic environment for 0.5 h, 1 h, 2 h, 4 h, 8 h, 18 h and 24 h. Protein levels of HIF-1α, HIF-2α in HCT116 and SW480 cells were assessed by western blotting. α-tubulin were used as an internal control. (**B**) Relative expression levels of c-Myc, HIF-1α, and HIF-2α protein over time are illustrated. All experiments were performed in triplicate.

Hypoxia inducible factors (HIFs) are key transcription factors mediating cellular responses to hypoxia. We investigated how chronic hypoxic stress affects the expression of HIF-1α and HIF-2α in HCT116 and SW480 cells. Over time (0.5, 1, 2, 4, 8, 18 and 24 h of hypoxia), the expression of HIF-1α was strongly induced at 2 h but significantly decreased in prolonged hypoxia. In contrast, HIF-2α was upregulated after 2 h of hypoxia and continued to increase over 24 h (Figure 2A). These results indicated that HIF-1α was induced acutely in hypoxia but that HIF-2α predominated over HIF-1α in colon cancer cells under chronic hypoxic conditions.

**Figure 3:**
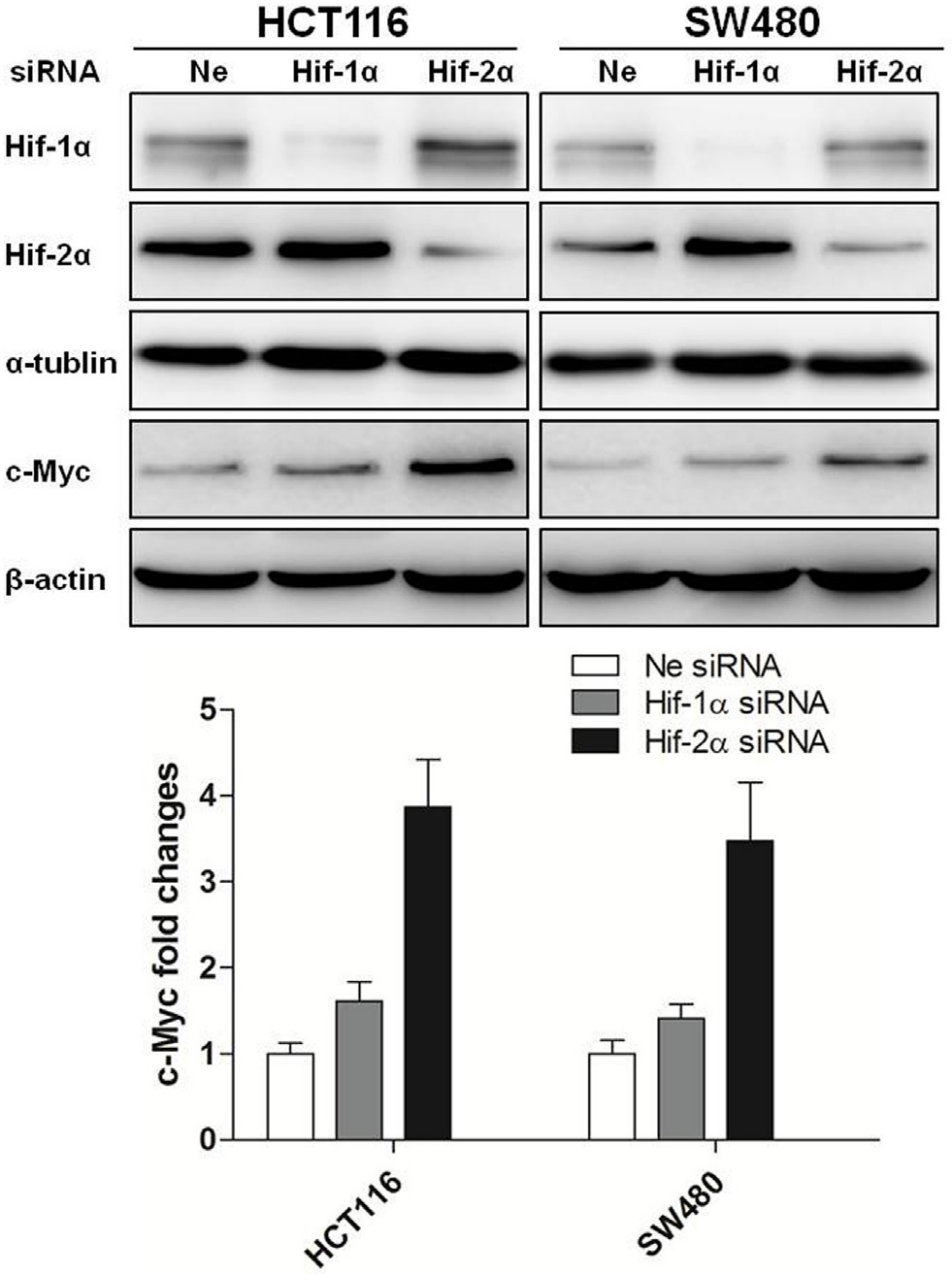
Regulation of c-Myc expression by HIF-2α in colon cancer cells The expression of HIF-1α, HIF-2α and c-Myc were measured by Western blotting after HCT116 and SW480 cells were transfected with siRNAs targeting HIF-1α, HIF-2α and then cultured under hypoxia for 24 h. Densitometry values are shown as fold change relative to negative control siRNA, which was normalized to 1. All experiments were performed in triplicate.

Interestingly, the changes in HIF-2α expression but not HIF-1α were inversely related to the expression levels of c-Myc protein over 24 h of hypoxia (Figure 2B), suggesting that HIF-2α may serve to repress c-Myc. To evaluate this interplay between HIFs and c-Myc, colon cancer cells (HCT116 and SW480) were transiently transfected with siRNA against HIF-1α or HIF-2α and incubated in hypoxia for 24 hours. Knockdown of HIF-2α reversed the downregulation of c-Myc in HCT116 and SW480 cells (3.87- and 3.48- fold change, respectively) induced by chronic hypoxia. In contrast, knockdown of HIF-1α only slightly increased the level of c-Myc (1.61- and 1.41- fold change, respectively) (Figure 3).

### c-Myc can regulate sensitivity to 5-FU under chronic hypoxic conditions

**Figure 4:**
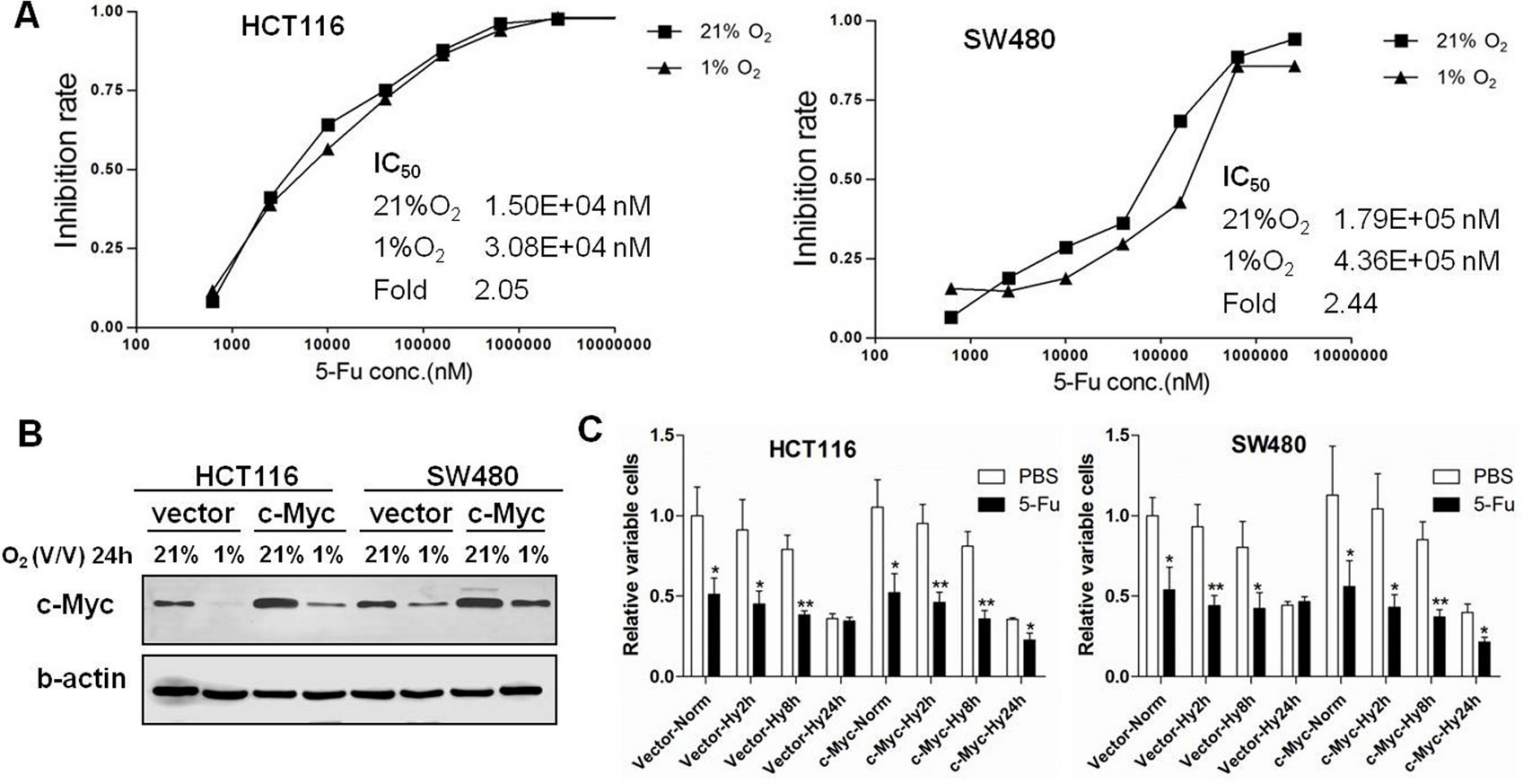
Hypoxic stress induces 5-FU chemoresistance in colon cancer cells (**A**) Growth inhibition rates were calculated at varying concentrations of 5-FU in HCT116 and SW480 cells under 21% O_2_ and 1% O_2_ for 24 h. The IC_50_ was calculated by modified Kou-type method. (**B**) The expression of c-Myc was detected by Western blotting after pCEP4 empty vector or pCEP4-c-Myc plasmid was transfected in HCT116 and SW480 cells and then cultured under normoxia or hypoxia for 24 h. (**C**) Relative cell viabilities were assessed by MTS after transfection of empty vector or c-Myc overexpression plasmid in HCT116 and SW480 cells under normoxic (Vector-Norm and c-Myc-Norm) or hypoxic (Vector-Hy2/8/24 h and c-Myc-Hy2/8/24 h) conditions after 2, 8 or 24 h. The value of empty vector transfection under normoxic conditions with PBS was normalized to 1. Asterisks indicate statistical significance (*P < 0.05, **P < 0.01, vs. PBS). All experiments were performed in triplicate.

To compare the chemosensitivity of 5-FU in normoxia and hypoxia, HCT116 and SW480 cells were treated with varying concentrations of 5-FU and cultured under 21% or 1% O_2_ for 24 hours. We observed that the IC_50_ of 5-FU in hypoxic conditions was higher than in normoxic conditions for both HCT116 and SW480 cells (2.05- and 2.44- fold change, respectively) (Figure 4A), indicating the development of resistance of 5-FU induced by chronic hypoxic stress.

To determine the role of c-Myc in the chemosensitivity of 5-FU in colon cancer cells in hypoxia, HCT116 and SW480 cells were transfected with empty control vector or c-Myc expression plasmid (Figure 4B), treated with 5-FU, and then cultured under normoxic or hypoxic conditions (2, 8 and 24 hours). 5-FU chemoresistance was observed when both cell lines were transfected with empty vector in chronic hypoxic conditions (24 hours), but stable overexpression of c-Myc in HCT116 and SW480 cells enhanced the chemosensitivity to 5-FU. In contrast, acute hypoxic (2 and 8 hours) stress did not influence the chemosensitivity of 5-FU in colon cancer cells regardless of c-Myc expression levels (Figure 4C). We further investigated the chemosensitivity to 5-FU after knockdown of c-Myc by targeted siRNA in colon cancer cells (Supplementary Figure S1A). Relative cell viabilities were higher in HCT116 and SW480 cells transfected with c-Myc siRNA and treated with IC_50_ concentration levels of 5-FU, indicating that knowledge of c-Myc increases sensitivity to 5-FU in colon cancer cells (Supplementary Figure S1B).

### c-Myc accelerates the G1/S cell cycle transition through regulation of cyclinD1 and CDK4/6

**Figure 5:**
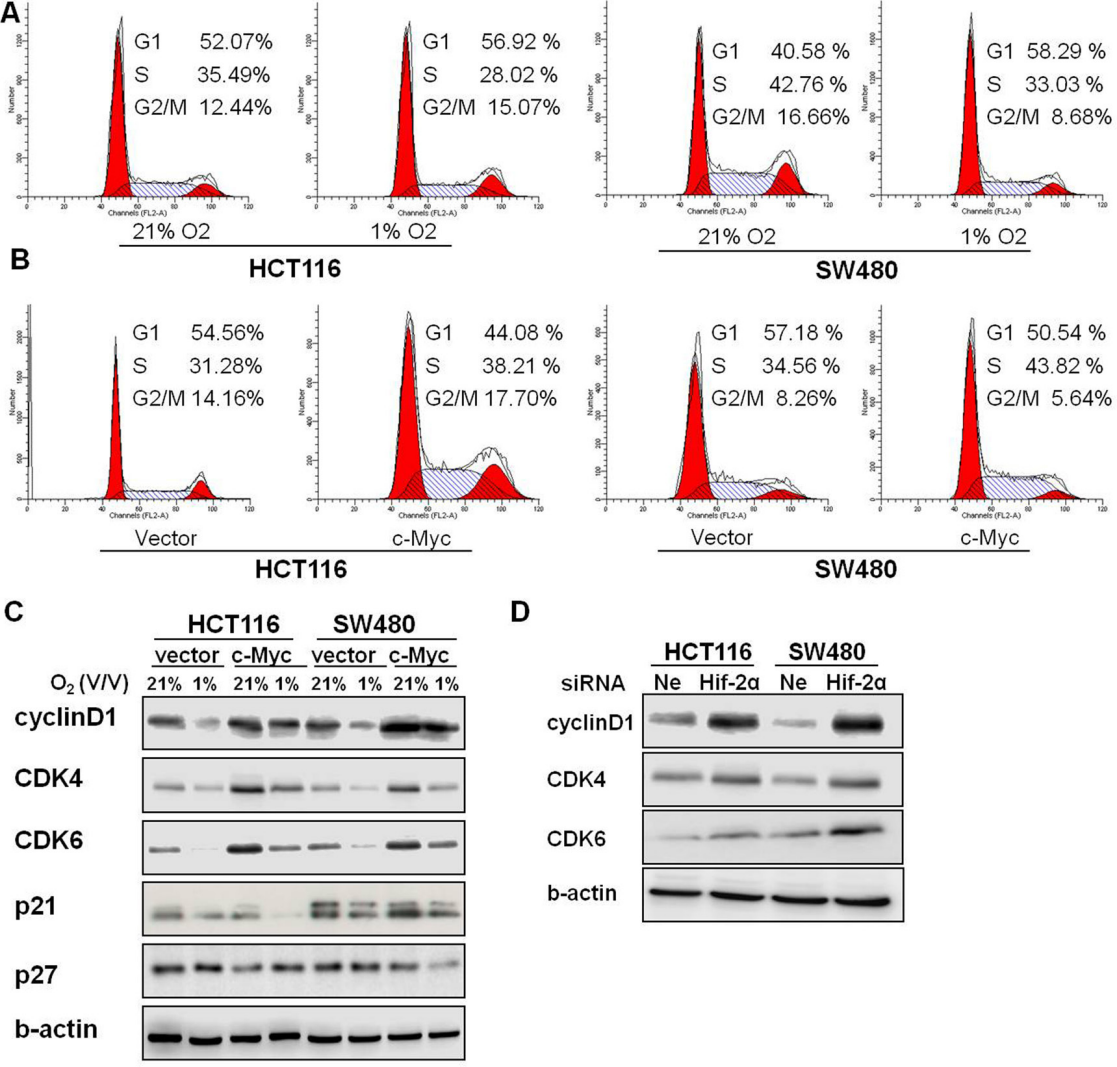
Overexpression of c-Myc altered the G1/S checkpoint in chronic hypoxia (**A**) Cell-cycle distributions were analyzed by flow cytometry in HCT116 and SW480 cells under 21% O_2_ and 1% O_2_. (**B**) HCT116 and SW480 cells overexpressing c-Myc were incubated in 1% O_2_, and cell-cycle distributions were analyzed by flow cytometry. (**C**) The expression of cyclin D1, CDK4, CDK6, p21, and p27 were assessed after the pCEP4-c-Myc plasmid was transfected in HCT116 and SW480 cells and then cultured under hypoxia for 24 h. (**D**) The expression of cyclinD1, CDK4 and CDK6 were assessed after transfection with HIF-2α siRNA in HCT116 and SW480 cells in hypoxia for 24 h. All experiments were performed in triplicate.

Inhibition of DNA replication in S phase is the primary mechanism of action of 5-FU. HCT116 and SW480 cells were sorted by flow cytometry and higher G1 phase and lower S phase populations were found in hypoxic conditions when compared to normoxia (Figure 5A). These results suggested that hypoxic stress could induce 5-FU chemoresistance by controlling G1/S arrest in colon cancer cells. We evaluated the cell cycle distribution of colon cancer cells transfected with c-Myc or control plasmids under hypoxia. The results demonstrated that a higher number of tumor cells entered S phase when c-Myc was overexpressed in hypoxia (Figure 5B). Collectively, these results suggest that c-Myc may regulate the chemosensitivity of 5-FU by altering the G1/S transition in hypoxia.

Cyclins, cyclin-dependent kinases (CDKs) and cyclin-dependent kinase inhibitors (p21, p27) regulate the G1/S transition. In order to clarify the mechanisms underlying c-Myc regulating of the cell cycle, we measured the expression of these cell cycle-related proteins after a c-Myc plasmid was introduced. The expression of cyclinD1, CDK4 and CDK6 were reduced by hypoxia in both HCT116 and SW480 cells, but forced expression of c-Myc partially reversed this. No consistent alterations were observed in p21 and p27 expression in HCT116 and SW480 cells (Figure 5C). To evaluate whether HIF-2α was also involved in the regulation of cell cycle proteins in chronic hypoxia, we measured the expression levels of cyclinD1 and CDK4/6 after knockdown of HIF-2α in HCT116 and SW480 cells. Silencing of HIF-2α increased the protein levels of cyclinD1, CDK4 and CDK6 (Figure 5D).

## DISCUSSION

Hypoxia is a critical component of the tumor microenvironment that influences tumor behavior. Hypoxia can be divided into acute and chronic phases, with variable biological and therapeutic consequences [13]. In large solid tumors, hypoxic stress can have a lasting impact [14]. Two key transcriptional regulators, HIF-1α and HIF-2α, play a pivotal role in the adaptive responses to hypoxia [4]. However, the relative contributions of these isoforms over time has not been well characterized [7]. Our results indicate that HIF-1α was more highly expressed than HIF-2α in CRC cells under acute hypoxia (within 2 hours). However, as the duration of hypoxia increased, HIF-2α levels gradually surpassed HIF-1α. The basis for this isoform switch is not fully understood [15].

The c-Myc oncogene is regulated by HIFs, and c-Myc is downregulated in low-oxygen regions of solid tumors [16]. We have demonstrated that HIF-2α appears to play an important role in downregulating c-Myc in chronic hypoxia in colon cancer cells. In our study, the transcriptional activity and protein levels of c-Myc were reduced in colon cancer cells in chronic hypoxia (24 h), indicating that the downregulation of c-Myc in chronic hypoxia was transcriptional. Consistent with the isoform specificity, knockdown of HIF-2α but not HIF-1α significantly increased the expression of c-Myc and its downstream target cyclinD1. The relationship between HIF and c-Myc is complex. It has been previously reported that HIF-2α can negatively regulate the expression of c-Myc in lung cancer cells [5]. However, Gordan *et al.* observed that HIF-2α can enhance the activity of c-Myc protein in renal cancer cells [7]. It should be noted that many of these studies were performed in the context of exogenously overexpressed protein. It is likely that the regulation of c-Myc by HIFs is cell type-specific and that there are multiple levels of regulation on the transcriptional and post-translational level. There are also other mechanisms that may contribute to c-Myc degradation in hypoxic conditions. For example, it has been shown that Fbw7 ubiquitin ligase and F-Box Protein Skp2 are involved in this process [17], but the specific effect of hypoxic stress on ubiquitination of c-Myc is unclear.

The implications of c-Myc downregulation in hypoxia are significant. C-Myc is a key regulator of the cell cycle. Most cells in hypoxia are blocked in G1 phase [18], rendering them resistant to agents like 5-FU that depend upon active DNA synthesis and replication. C-Myc may then play a key role in determining chemosensitivity to 5-FU, which is most effective at killing cells that are rapidly dividing [19]. Interestingly, in CRC patients receiving postoperative 5-FU chemotherapy, positive c-Myc status has been associated with a better prognosis [12]. In a study by Guichard et al. [20], a greater number of CRC cells were recruited into S phase when irinotecan was added, and the combined use of irinotecan and 5-FU sharply decreased the IC_50_ concentration of 5-FU.

C-Myc has many complex functions. For example, c-Myc is a regulator of both cell cycle progression and apoptosis. Amplification of c-Myc is detected in many cancers [10]. As a pivotal transcription factor, c-Myc has a large downstream regulatory network [21]. Its carboxyl terminus contains a basic region helix-loop-helix leucine zipper motif that interacts with a consensus enhancer binding motif (E-box: 5′-CACGTG-3′) in the promoter region of target genes [22] or a consensus repressor element (5′- CCAGACC-3′) [23]. C-Myc positively regulates the expression of CDK4 and CDK6 [24], but there is some controversy on the regulation of cyclinD1 [25, 26]. Our results have shown that c-Myc can lead to reaccumulation of cyclinD1, CDK4 and CDK6 in hypoxia, and this potentially explain the G1/S switch induced by c-Myc in colon cancer cells in a low-oxygen environment. In addition, silencing of HIF-2α induced the expression of cyclinD1, CDK4 and CDK6 in hypoxia, suggesting a key regulatory effect of the HIF-2α/c-Myc axis in the cell cycle.

In summary, we have demonstrated that HIF-1α and HIF-2α are differentially regulated by hypoxia in CRC cells. The long-term induction of HIF-2α but not HIF-1α appears to suppress c-Myc and contribute to cell cycle arrest and resistance to 5-FU chemotherapy. Strategies to reverse this regulatory network may enhance treatment options to target cells in this refractory environment.

## MATERIALS AND METHODS

### Cell culture

HCT116 and SW480 cells were obtained from American Type Culture Collection (Manassas, USA). HCT116 cells were cultured in McCoy's 5A medium (Invitrogen, Carlsbad, CA) and incubated at 5% CO_2_, 37°C and 95% humidity; SW480 cells were cultured in Leibovit's L-15 medium (Invitrogen) and incubated at 5% CO_2_, 37°C and 95% humidity. Both of these media were supplemented with 10% fetal bovine serum (HyClone, Ogden, UT) and 100 U/ml antibiotics (penicillin–streptomycin, Invitrogen). Hypoxic conditions were achieved by culturing cells in a sealed hypoxia chamber (Billups-Rothenberg, Del Mar, CA) with mixed gas containing 1% O_2_.

### Western blotting

Total cellular protein was extracted using RIPA lysis buffer (Cell Signaling Technology Inc., Danvers, USA) supplemented with 1nM PMSF (Cell Signaling Technology Inc.). Lysates were resolved on SDS-PAGE gel and transferred to PVDF membranes (Millipore, Bedford, USA). The blots were probed with c-Myc (1:1000, Cell Signaling Technology Inc.), p-c-Myc (1:1000, Cell Signaling Technology Inc.), HIF-1α (1:1000, BD Biosciences, Franklin Lakes, USA), HIF-2α (1:1000, Abcam, Cambridge, USA), cyclinD1 (1:1000, Cell Signaling Technology Inc.), CDK4 (1:500, Cell Signaling Technology Inc.), CDK6 (1:1000, Cell Signaling Technology Inc.), p21 (1:1000, Cell Signaling Technology Inc.), p27 (1:1000, Cell Signaling Technology Inc.), α-tubulin (1:1000, Cell Signaling Technology Inc.) or β-actin (1:1000, Cell Signaling Technology Inc.) antibodies. The blots were visualized using standard techniques for chemiluminesence.

### Quantative real-time PCR (qPCR)

Total RNA was extracted from colon cancer cells with Trizol reagent (Invitrogen). cDNA was produced from 1 μg RNA using M-MLV reverse transcriptase (TaKaRa, Otsu, Japan) with random primers (TaKaRa). The expression of β-actin in each sample was used as an internal control. qPCR was performed using SYBR Green Master Mix Kit (Takara) in an ABI 7500 PCR system (Thermo Fisher). Primers used for PCR reactions were as follows, c-Myc-F: GAGTTTCATCTGCGACCCG, c-Myc-R: GCTGCCGCTGTCTTTGC; β-actin-F: CACCAACTGGGACGACAT, β-actin-R: AGCACAGCCTGGATAGCA.

### Plasmid construction and cell transfection

Full-length c-Myc cDNA was cloned and ligated to the mammalian expression vector pCEP4 (Invitrogen) with *KpnI* and *BamHI* restriction enzyme sites. HCT116 and SW480 cells were transfected with pCEP4 empty vector or pCEP4-c-Myc plasmid using Fugene HD Reagent (Promega, Madison, USA). Two days later, those cells were subcultured and selected by 200 μg/ml hygromycin (Invivogen, San Diego, USA) for another 14 days to generate stable c-Myc overexpressing cell lines. For siRNA-mediated gene knockdown, HCT116 and SW480 cells were transfected with negative control siRNA, HIF-1α targeted siRNA, HIF-2α targeted siRNA or c-Myc targeted siRNA (Invitrogen) using Lipofectamine RNAiMAX Reagent (Invitrogen). Six hours after transfection, fresh culture medium was added and cells were placed in a 21% O_2_ or 1% O_2_ incubator for 24 hours before further measurement.

### Luciferase activity assay

HCT116 and SW480 cells were seeded in 24-well plates. At 50–60% confluence, cells were cotransfected with 0.04 μg pGL3-c-Myc-promoter plasmid containing 2.0 kb nucleotide sequence at the upstream region of c-Myc and 0.004 μg pRL-TK vector (Promega) containing renilla luciferase as an internal control using Fugene HD. The cells were then cultured under normoxic or hypoxic conditions for 24 h. 200 μl of passive reporter lysis buffer (Promega) was added to cells. Luciferase activity was analyzed by the dual-luciferase reporter assay system according to the manufacturer's protocols (Promega).

### Protein stability assays

HCT116 and SW480 cells were treated with 10 μg/ml cycloheximide and then cultured in hypoxic conditioning for 2 or 8 h. c-Myc protein levels were measured by western blotting at the end of the hypoxic period.

### Calculation of IC_50_

HCT116 and SW480 cells were treated with 5-FU at a geometric concentration gradient of 0.625, 2.5, 10, 40, 160, 640, and 2560 μM and cultured in 21% or 1% O_2_ for 24 h. Cold, non-radioactive MTS reagent was added and incubated at 37°C for 1 h. Relative cell viabilities were measured according to the absorbance at 490 nm by a spectrophotometer. The values of those wells containing only culture medium without cells seeded were regarded as blank, and those containing cells without 5-FU were recognized as control. Growth inhibition rates at each concentration were calculated according to the formula: (OD value of control group – OD value of 5-FU group) / (OD value of control group – OD value of blank group). Then IC_50_ of 5-FU was calculated by modified Kou-type method as previously described [27].

### 5-FU cytotoxicity assay

HCT116 and SW480 cells were treated with 5-FU at the previously established IC_50_ concentration and cultured in normoxia or hypoxia for 2, 8 or 24 h. The total 5-FU exposure time was 24 h, and cell viabilities were assessed by MTS assay.

### Flow cytometry (FCM) analysis

After hypoxic treatment, cells were harvested and cell cycle distribution was assessed with the cell cycle staining kit (Multisciences, Hangzhou, China). Briefly, 10^6^ cells were washed in PBS and resuspended with 1 ml DNA staining solution. After incubation for 30 min at room temperature in the dark, cells were sorted by flow cytometry (BD Biosciences, Franklin Lakes, USA). Then cell cycle distribution was analyzed with the ModFit LT software (Phoenix, USA).

## SUPPLEMENTARY MATERIALS


