# Supplementary material for: c-Myc is regulated by HIF-2α in chronic hypoxia and influences sensitivity to 5-FU in colon cancer
Source: Oncotarget. 2016 Oct 26;7(48):78910–7. doi: 10.18632/oncotarget.12911 (PMC5346686; doi:10.18632/oncotarget.12911)
Supplement: Supplementary file 1 [file oncotarget-07-78910-s001.pdf]

c-Myc is regulated by HIF-2α in chronic hypoxia and influences sensitivity to 5-FU in colon cancer

Supplementary Materials

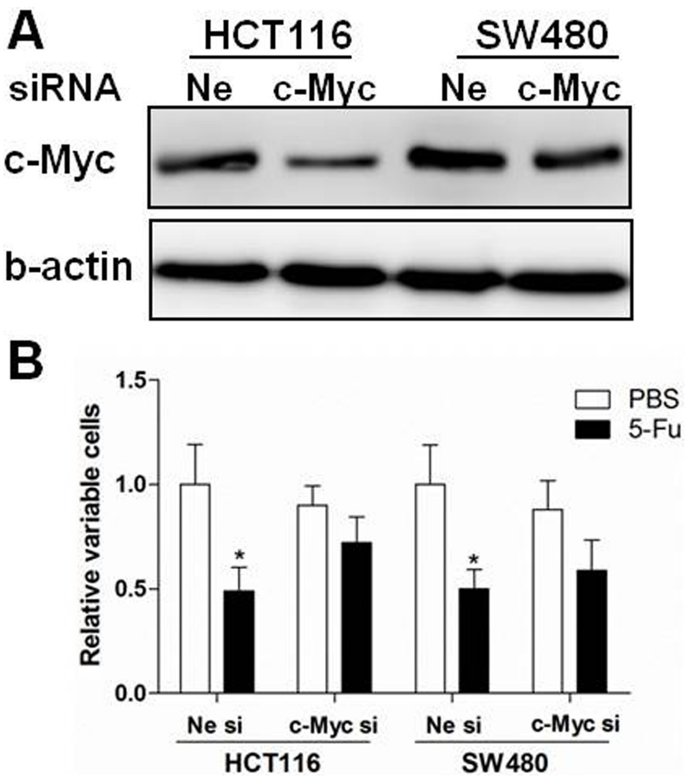

Supplementary Figure S1: Knockdown of c-Myc impairs chemosensitivity to 5-FU in colon cancer cells. (**A**) The expression of c-Myc was measured by Western blotting after transfection with a c-Myc targeted siRNA in HCT116 and SW480 cells. (**B**) Relative cell viabilities were assessed by MTS after transfection of negative control siRNA or c-Myc targeted siRNA in HCT116 and SW480 cells. Asterisks indicate statistical significance.
